# Analysis of the role of PrrA, PpsR, and FnrL in intracytoplasmic membrane differentiation of *Rhodobacter sphaeroides* 2.4.1 using transmission electron microscopy

**DOI:** 10.1007/s11120-013-9944-9

**Published:** 2013-10-22

**Authors:** Yana Fedotova, Jill Zeilstra-Ryalls

**Affiliations:** Department of Biological Sciences, Bowling Green State University, Bowling Green, OH 43403 USA

**Keywords:** *Rhodobacter sphaeroides*, Intracytoplasmic membrane formation, FnrL, Prr, PpsR, Oxygen control

## Abstract

Oxygen dictates the catabolic “lifestyle” of *Rhodobacter sphaeroides*. When it is present, the bacteria are fully equipped for aerobic respiration. When it is absent, the cells outfit themselves to make use of energy-gathering options that do not require oxygen. Thus, while respiring on alternate electron acceptors in the absence of oxygen even in the dark, the cells are fully enabled for phototrophy. PrrA, PpsR, and FnrL are global regulatory proteins mediating oxygen control of gene expression in this organism. For each of these, regulon members include a subset of a cluster of genes known as the photosynthesis genes, which encode the structural proteins and enzymes catalyzing biosynthesis of the pigments of the light-harvesting and reaction center complexes. The complexes are housed in a specialized structure called the intracytoplasmic membrane (ICM). Although details are emerging as to the differentiation process leading to fully formed ICM, little is known of necessary regulatory events beyond changes in photosynthesis gene transcription. This study used transmission electron microscopy toward gaining additional insights into potential roles of PrrA, PpsR, and FnrL in the formation of ICM. The major findings were (1) the absence of either PrrA or FnrL negatively affects ICM formation, (2) the lack of ICM in the absence of PrrA is partially, but not fully reversed by removing PpsR from the cell, (3) unlike *R. sphaeroides*, ICM formation in *Rhodobacter capsulatus* does not require FnrL. New avenues these findings provide toward identifying additional genes involved in ICM formation are discussed.

## Introduction

The cytoplasmic membrane (CM) plays a universal role in cells of all three domains of life. This semipermeable barrier isolates the cytoplasm from the external environment, but environmental changes can result in changes in gene expression that lead to alterations in composition and concentration of both lipids and proteins. The membrane can also undergo regulated restructurings that are critical to cell function. In eukaryotic cells, these events, such as those triggered by phagocytosis and cell motility, are commonplace (Lippencott and Li 2000). However, among bacteria, only a few such restructurings have been described, and are thus far limited to the α-proteobacteria.

One such restructuring event is the differentiation of the *Rhodobacter sphaeroides* CM leading to the formation of the intracytoplasmic membrane (ICM) that houses the photosynthesis system of these bacteria (Chory et al. 1984), consisting of the pigment–protein complexes of the reaction center (RC) and the two light-harvesting complexes, LHI and LHII. Our present understanding of the composition and development of *R. sphaeroides* ICM has been comprehensively reviewed recently (Niederman 2013). As is appropriate for (facultative) anoxygenic photosynthesis, ICM formation is induced by lowering oxygen tensions, and in *R. sphaeroides* wild type strain 2.4.1 three DNA binding proteins that mediate oxygen control of phototrophic growth and/or PS genes (genes that code for the structural proteins, and the enzymes that synthesize the photopigments of the photosynthetic apparatus) are known. *P*hotosynthesis *r*esponse *r*egulatory protein *A* (PrrA) is the DNA binding regulatory protein of a redox-responsive two-component regulatory system (Eraso and Kaplan 1994, 1995). A functional *prrA* gene is required for phototrophic growth of *R. sphaeroides* 2.4.1 (Eraso and Kaplan 1994). *P*hoto*p*igment *s*uppressor protein *R* (PpsR) is a transcription repressor of PS genes under aerobic conditions that was initially characterized by Penfold and Pemberton (1994). Its most important role is thought to be preventing the coincidence of Bchl *a* in the presence of oxygen and light (Moskvin et al. 2005), which can create a lethal situation through the production of reactive oxygen species. *F*umarate-*n*itrate reductase *r*egulator-type protein *L* (FnrL) is the *R. sphaeroides* homolog of the global anaerobic regulatory Fnr protein of *E. coli* (Zeilstra-Ryalls and Kaplan 1995). Unlike PrrA, FnrL is essential for all anaerobic growth of *R. sphaeroides* 2.4.1, which includes anaerobic growth in the dark with the alternate electron acceptor dimethyl sulfoxide (DMSO) and anaerobic growth in the light (Zeilstra-Ryalls and Kaplan 1995).

Until now, the roles of these regulators in ICM formation have been extrapolated from investigations of the genes they regulate, together with spectral analysis of pigments and pigment–protein complexes. Here, we present our novel findings regarding these transcription factors based on a direct examination of the ultrastructure of wild type versus mutant cells missing one or more of the DNA binding proteins, and also describe new directions they provide for investigating this membrane restructuring event.

## Materials and methods

### Bacterial strains, plasmids, and growth conditions

*Rhodobacter sphaeroides* and *Rhodobacter capsulatus* strains used in this study are listed in Table 1, together with their relevant characteristics and sources. In all cases, Sistrom’s succinate minimal medium A (Sistrom 1960) was used for growth of *R. sphaeroides*. *R. capsulatus* strains were grown in Sistrom’s succinate minimal medium A supplemented with 0.4 % fructose. Low-oxygen growth was achieved by inoculation of *R. sphaeroides* or *R. capsulatus* into 100 ml of medium in 250 ml Erlenmeyer flasks that were incubated at 30 °C in a New Brunswick gyratory shaking water bath (model G76) at 90 rpm. Anaerobic growth was performed by inoculation of screw-capped tubes completely filled with medium that was supplemented with 0.1 % yeast extract and 60 mM dimethyl sulfoxide as alternate electron acceptor.

**Table 1:** *Rhodobacter* strains used in this study

Strain	Relevant characteristics	Reference or source
*R. sphaeroides*		
2.4.1	Wild type	Sistrom (1960)
BR107	Δ*prrA*::*loxP*	Ranson-Olson and Zeilstra-Ryalls (2008)
PRRBCA2	Δ(BspEII-Tth111I)*prrBAC*::Tp^R^	Oh et al. (2000)
PRRA1	*prrA*(PstI)::ΩSp^R^/St^R^	Eraso and Kaplan (1995)
PRRA2	Δ(BstBI-PstI)*prrA*::ΩSp^R^/St^R^	Eraso and Kaplan (1995)
PPS1	*ppsR*::ΩKn^R^	Gomelsky and Kaplan (1997)
RPS1	*ppsR*::ΩKn^R^ *prrA*::ΩTp^R^	Moskvin et al. (2005)
JZ1678	Δ*fnrL*::ΩKn^R^	Zeilstra-Ryalls and Kaplan (1995)
*R. capsulatus*		
2.3.1	Wild type	American Type Culture Collection
SB1003	Spontaneous Rif^R^ prototrophic derivative of 2.3.1	Yen and Marrs (1976)
RGK295	Δ*fnrL*::Kn^R^ derivative of SB1003	Zeilstra-Ryalls et al. (1997)
RGK296	Δ*fnrL*::Kn^R^ derivative of SB1003; Kn^R^ in opposite direction to RGK295	Zeilstra-Ryalls et al. (1997)

### Transmission electron microscopy (TEM)

The preparation of grids has been described previously (Fedotova 2010). This involved fixing cells in Karnovsky’s fixative solution (Karnovsky 1965), staining them with osmium tetroxide (Electron Microscopy Sciences, Inc. Hatfield, PA), and then dehydrating them. The dehydrated cells were infiltrated with Spurr’s low viscosity embedding medium (Spurr 1969), and the mixture was polymerized to form blocks of embedded cells which were sectioned with a Sorvall Porter-Blum MT-2 Ultra Microtome using a diamond knife (Delaware Diamond Knives, Inc., Wilmington, DE) to sections with thicknesses of approximately 70 nm. The sections, transferred onto copper-coated 300 mesh square carbon grids, were first stained with an alcoholic solution of 2 % (w/v) uranyl acetate and then with Reynolds lead citrate stain (Reynolds 1963). The thinly sectioned cells were visualized using a Zeiss EM-10 transmission electron microscope at 60 kV accelerating potential, and images were captured onto Kodak 4489 film (Rochester, NY).

### Spectral analysis of membrane fractions and quantitation of pigments

Protein synthesis was halted by the addition of chloramphenicol solution (20 mg/ml in 95 % ethanol) to a final concentration of 1.5 % (v/v) to the cultures which were then chilled on ice. The cells were pelleted at 2,688×*g* for 10 min at 4 °C, and then the cell pellet was resuspended in 5 ml of 0.1 M sodium phosphate buffer, pH 7.7. Immediately prior to lysis, a protease inhibitor cocktail (Sigma Chemical Co., St. Louis, MO) was added (100 μl/50 ml of culture). The cells were lysed by passaging them through a French pressure cell at 700 psi. Insoluble debris was pelleted by centrifugation for 20 min at 21,952×*g* at 4 °C. Spectra were recorded between wavelengths of 950–350 nm using a Hitachi U-2010 UV/Vis Spectrophotometer (Hitachi High Technologies America, Inc., Schaumburg, Illinois). The Bchl *a* levels in the photosynthetic pigment–protein complexes were calculated from the spectral data using the method of Meinhardt et al. (1985).

### Protein concentration determinations

Protein concentrations were determined using the Pierce BCA Protein Assay Reagent (Pierce, Rockford, IL). Bovine serum albumin was used as a standard.

## Results

### Ultrastructure of *R. sphaeroides* wild type 2.4.1 and *prr* mutant bacteria

The Prr redox-responsive two-component system is composed of the PrrB membrane-localized sensor protein and the PrrA cytoplasmic DNA binding regulatory protein. A third membrane-localized protein, PrrC, is thought to communicate the redox signal, the nature of which is as yet unknown, to PrrB. These features, and other details about the regulatory system and its impact on gene transcription in response to changes in oxygen availability have been reviewed recently (Gomelsky and Zeilstra-Ryalls 2013). Although PrrA^−^ mutants cannot grow phototrophically, their respiratory capacity is apparently unaffected, and they can grow in the dark both aerobically and anaerobically using dimethyl sulfoxide (DMSO) as alternate electron acceptor. Since the absence of oxygen is necessary and sufficient to induce transcription of photosynthesis genes, it was possible to establish that cells lacking *prrA* have no detectable photosynthesis pigment–protein complexes by growing mutant bacteria under those permissive anaerobic–dark conditions (Eraso and Kaplan 1994). To explore the consequences for ICM formation directly, the ultrastructure of bacteria having a null mutation in *prrA* and also that are deleted of all three *prr* genes, *prrA*, *B*, and *C* was examined by TEM.

**Fig. 1:**
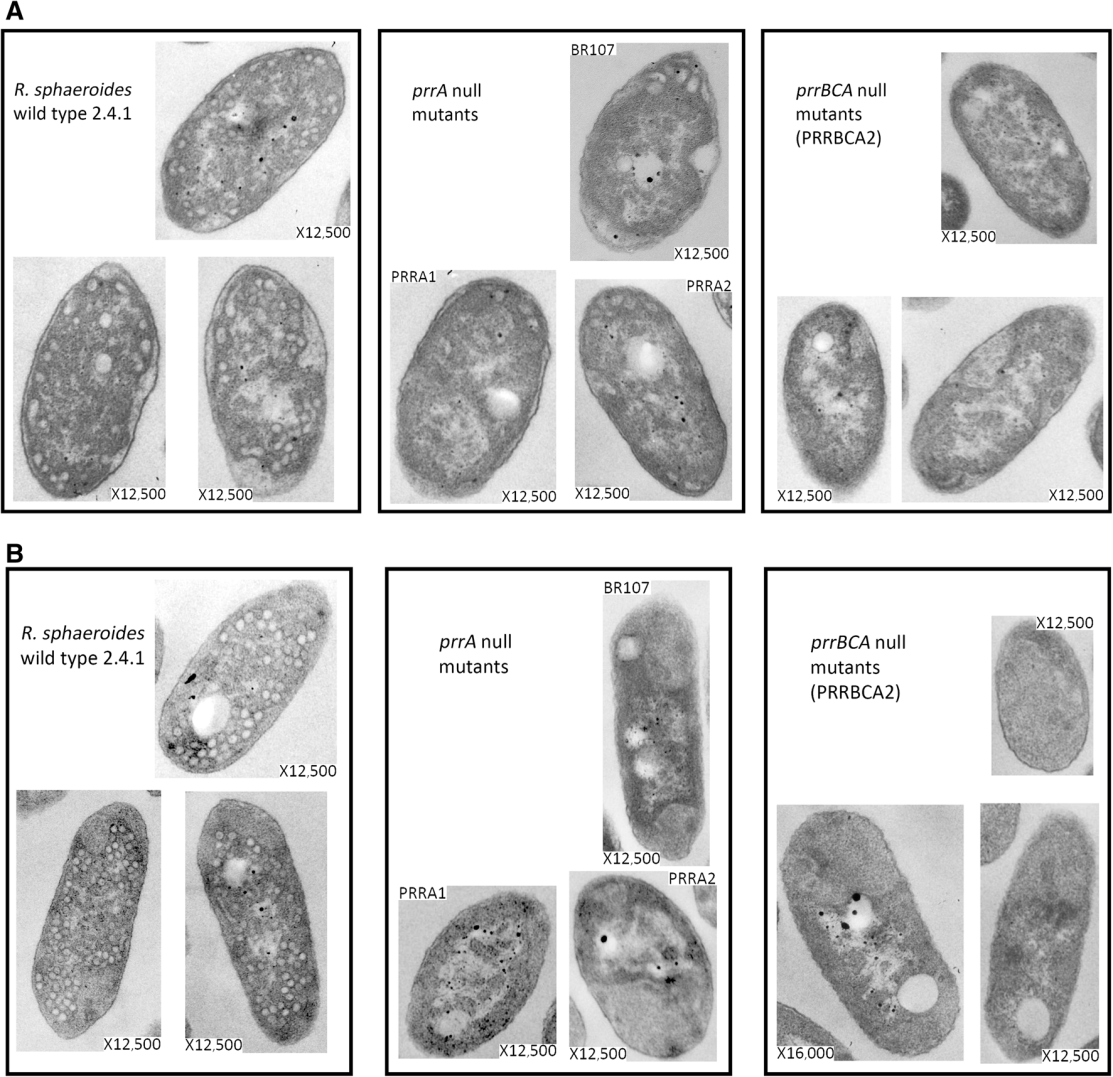
TEM of *R. sphaeroides* wild type 2.4.1, *prrA* ^−^ mutant, and *prrBCA* ^−^ mutant bacteria. Micrographs are of thin sections of cells cultured under **A** low-oxygen conditions or **B** anaerobic–dark conditions, with DMSO as alternate electron acceptor. The strains used are as explained in the legends, and details are provided in Table 1

Thin sections of cells cultured under both low-oxygen and anaerobic–dark with DMSO conditions were examined using TEM (Fig. 1). Fully developed ICM was observed in thin sections of the wild type 2.4.1 cells that had been cultured under either condition. For those mutants in which only the *prrA* gene is defective, strains PRRA1, PRRA2, and BR107 (Table 1), a low number, on average 5–10/cell, of ICM-like structures that are located at the cell poles were present in the thin sections of cells cultured under low-oxygen (Fig. 1A). No such structures were observed in the thin sections of *prrA* null mutant bacteria that had been grown anaerobically in the dark (Fig. 1B). ICM-like structures were also not observed among the sections of PRRBCA2 cells (Table 1) grown under low- (Fig 1A) or no oxygen (Fig. 1B) conditions. These results establish for the first time a phenotypic difference between cells that lack the response regulator alone versus cells that are missing the entire signal transduction system.

**Fig. 2:**
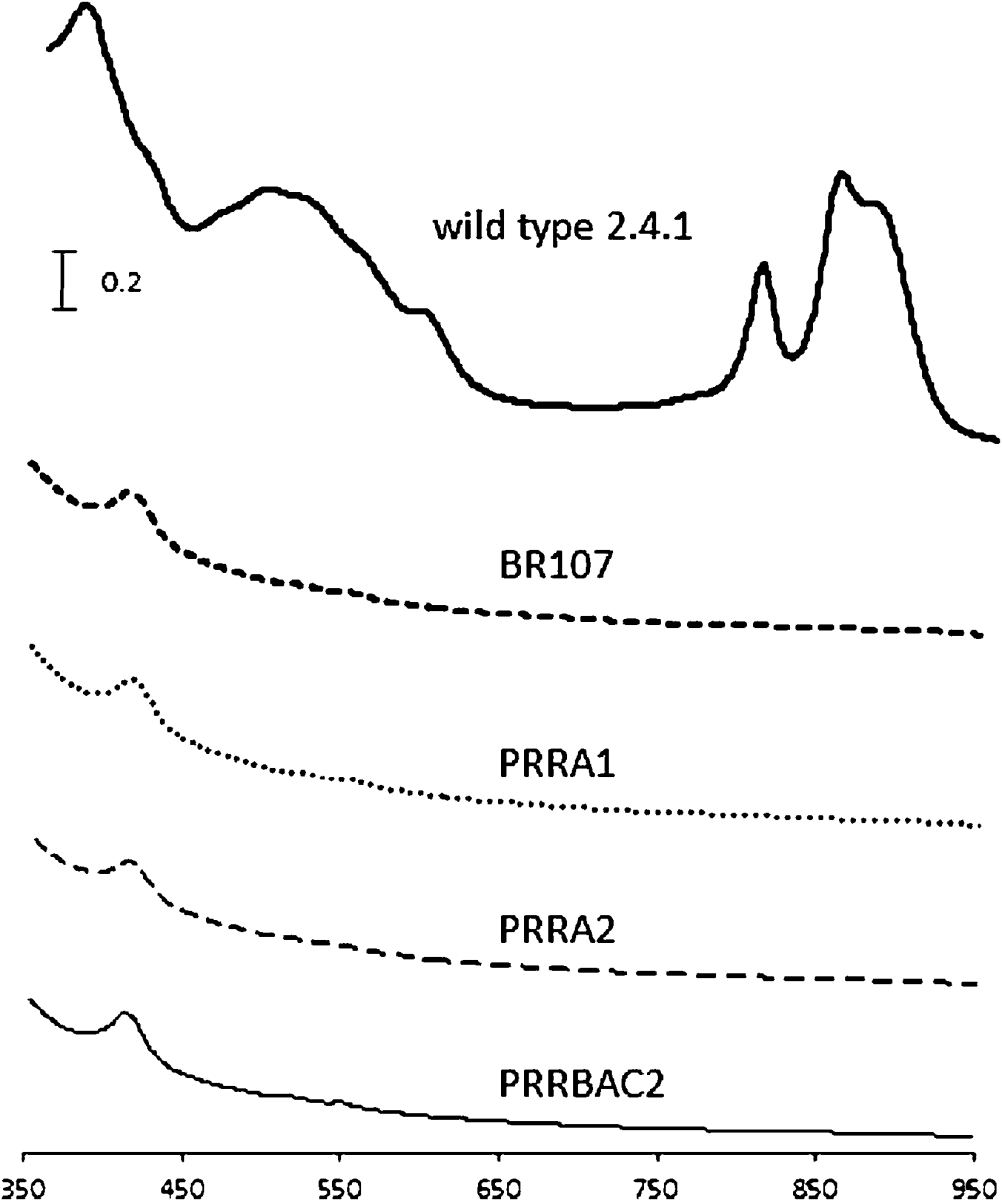
Spectral analysis of crude lysates of *R. sphaeroides* wild type 2.4.1, *prrA* ^−^ mutant, and *prrBCA* ^−^ mutant bacteria grown under low-oxygen conditions. The spectra correspond to lysates of the strains indicated, and were generated using samples having equivalent concentrations of total protein (1.3 mg/ml). Details regarding the strains are provided in Table 1. The peaks near 420 nm in the spectra of the mutant strain samples can be attributed to cytochrome Soret bands, mostly obscured in the spectrum of the wild type 2.4.1 sample

Transcriptomic profiling, accompanied by proteomic analysis of bacteria lacking PrrA has been performed for cells grown under anaerobic–dark conditions (Eraso et al. 2008). These analyses demonstrated that, in the absence of PrrA, transcription of photosynthesis genes is severely diminished, and for some among them it is to the degree that the protein products are completely undetectable. This includes structural proteins of RC (PufM and L) and LHI (PufA) and several enzymes required for production of photo-pigments (CrtA, E, I and BchD, H, N, and M). However, there are no corresponding data available for cells grown under low-oxygen conditions. The presence of ICM-like structures in the *prrA* null mutant bacteria raised the question as to whether or not the membranes contained any pigment–protein complexes. Spectral analysis of samples prepared from the same culture used for TEM indicated that the amounts of the pigment–protein complexes were below detectable levels in all the *prr* mutants cultured under low-oxygen conditions, and no differences between PrrA^−^ versus PrrBCA^−^ mutant bacteria were indicated using this method (Fig. 2). Therefore, the structural differences between the PrrA^−^ mutants versus the PrrBCA^−^ mutant in the presence of limited oxygen have only become apparent from the physical examination performed here using TEM.

### Ultrastructure of *R. sphaeroides* wild type 2.4.1, *ppsR* mutant, and *ppsRprrA* mutant membranes

PpsR has been called a “master” regulator of photosystem development (Moskvin et al. 2005), and disabling *ppsR* leads to the expression of photosynthesis genes in the presence of oxygen. Thus, cells lacking PpsR are genetically extremely unstable under aerobic conditions (Gomelsky and Kaplan 1997). The activity of PpsR is controlled by interactions with the anti-repressor protein AppA (reviewed in Gomelsky and Zeilstra-Ryalls 2013). Recent studies have shown that transcription of the *appA* gene is PrrA-dependent. They also indicate that PrrA appears to affect interactions between AppA and PpsR, which in turn influences the activity of PpsR. The consequences of this regulatory complexity are made apparent by virtue of the fact that, although phototrophic growth is abolished in *prrA* null mutant bacteria, bacteria lacking both PrrA and PpsR can grow phototrophically (Gomelsky et al. 2008). The status of either *ppsR* ^−^ or *ppsR* ^−^ *prrA* ^−^ mutant bacteria with respect to ICM formation has not been directly determined. In order to do so, TEM was used to examine the ultrastructure of cells grown under inducing anaerobic (dark) conditions that do not exert selective pressure for suppressor mutations that compensate for the absence of PpsR.

**Fig. 3:**
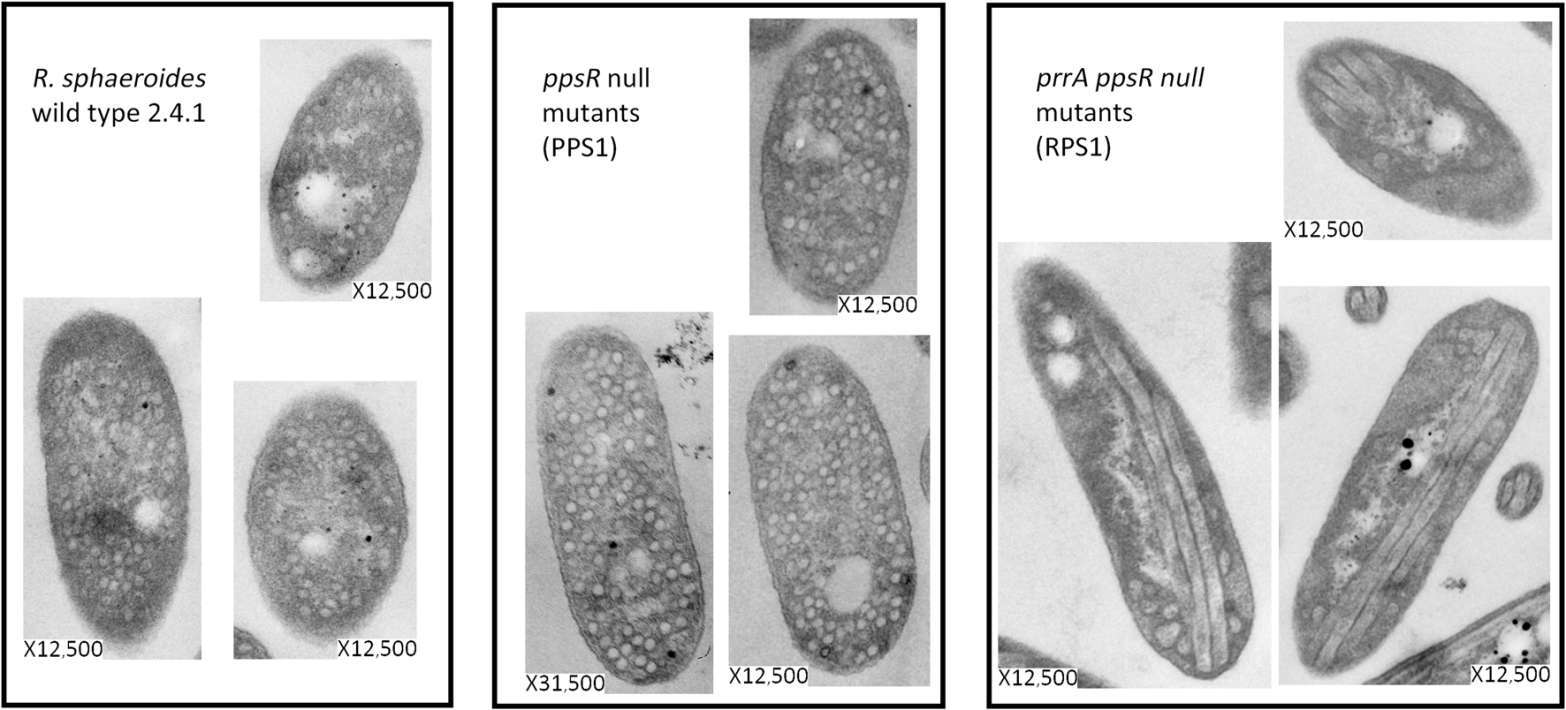
TEM of *R. sphaeroides* wild type 2.4.1, *ppsR* ^−^ mutant, and *prrA* ^−^ *ppsR* ^−^ mutant bacteria that had been cultured under anaerobic–dark conditions with DMSO as alternate electron acceptor. The strains used are as explained in the legends, and details are provided in Table 1

ICM formation was apparently not affected by the absence of PpsR, as the ultrastructure of the PPS1 (Table 1) mutant cell membrane appears similar to that of wild type bacteria (Fig. 3). This was to be expected, since PpsR functions as a repressor of PS genes under aerobic conditions, and *ppsR* null mutant bacteria grow normally under phototrophic conditions.

Since PrrA is thought to be necessary for the inactivation of PpsR (Moskvin et al. 2005; Gomelsky et al. 2008), the *ppsR* ^−^ *prrA* ^−^ double mutant strain RPS1 (Table 1) should have normal ICM. However, long, tubular-shaped ICM was found to be a prominent feature of the cells (Fig. 3). Evidently, despite the abnormal appearance of the ICM, the photosynthesis machinery is nevertheless at least somewhat operational as the cells can grow phototrophically, although their growth is considerably slower than wild type (Moskvin et al. 2005).

Spectral analysis of RPS1 has previously been reported (Moskvin et al. 2005; Gomelsky et al. 2008). The data indicate that the LHII antenna complexes are severely diminished relative to the wild type. The correlation between the reduction or lack of LHII and the presence of tubular structures has been noted by others (Kiley et al. 1988; Hunter et al. 1988; Sabaty et al. 1994; Siebert et al. 2004). But we believe this is the first report of such aberrant structures in regulatory gene mutants. Importantly, the available information regarding regulation of PS gene expression by PrrA and PpsR does not explain why LHII is absent while LHI and RC are present (Gomelsky et al. 2008). It implies that other genes necessary for proper ICM development, such as assembly factors required for LHII formation, are also inappropriately (not) expressed in the absence of PrrA and PpsR.

### Ultrastructure of *R. sphaeroides* and *R. capsulatus* wild type and *fnrL* mutant bacteria

FnrL belongs to the Fnr–Crp protein family (Zeilstra-Ryalls and Kaplan 1995). All members are characterized by the presence of an effector domain located within the N-terminal region and a DNA binding domain located within the C-terminal region. For FnrL, the effector domain is thought to contain an oxygen-labile 4Fe-4S cluster whose presence is required for the protein to be properly configured for DNA binding. Thus, the protein regulates gene transcription when oxygen is limiting. While FnrL is essential for all anaerobic growth of *R. sphaeroides* 2.4.1, both in the light and in the dark with DMSO (Zeilstra-Ryalls and Kaplan 1995), the reason for this is not yet resolved (detailed in Gomelsky and Zeilstra-Ryalls 2013).

**Fig. 4:**
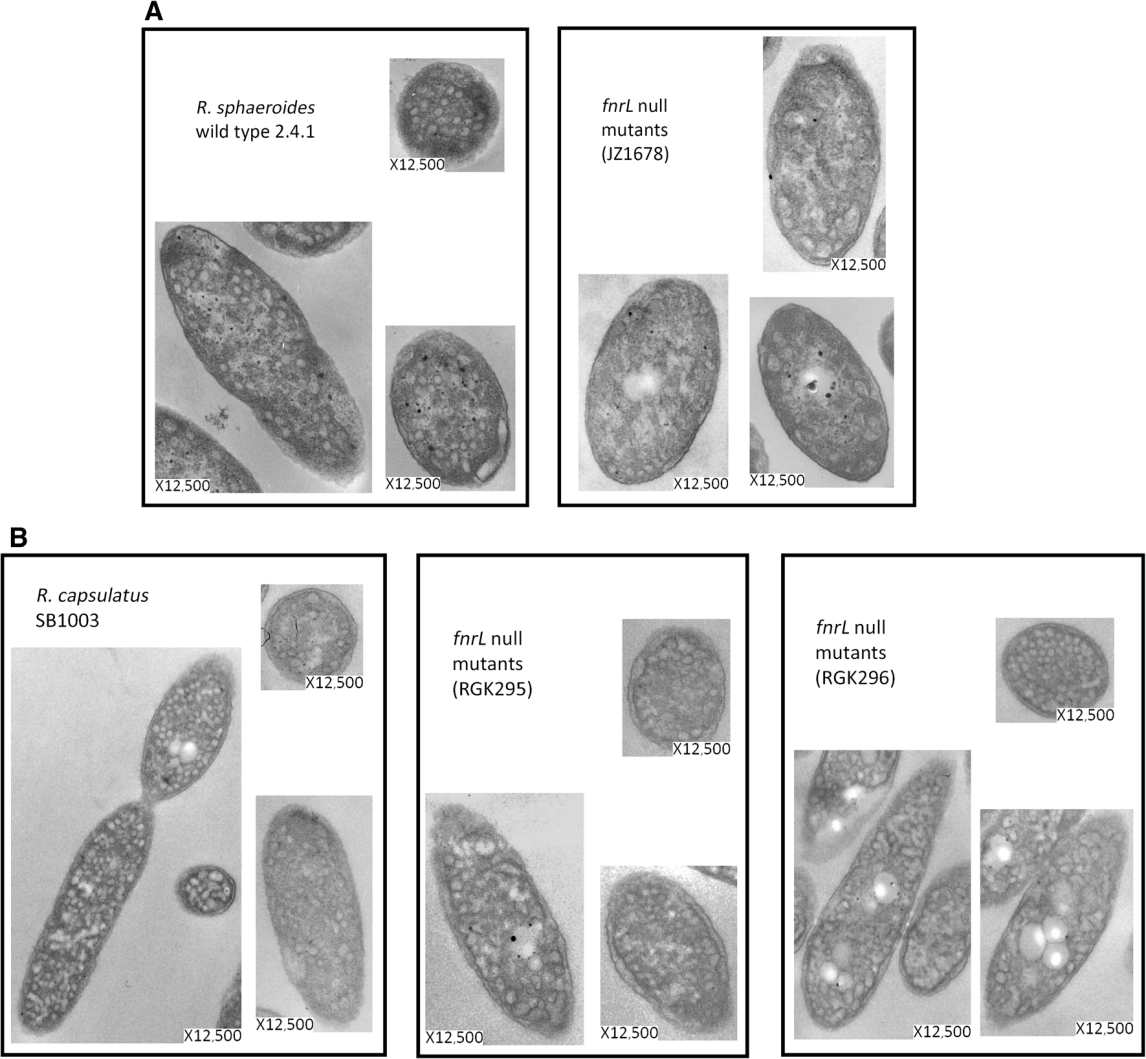
TEM micrographs of thin sections of wild type and mutant strains of *R. sphaeroides* (**A**) and *R. capsulatus* (**B**) bacteria that had been cultured under low-oxygen conditions. The strains used are as explained in the legends, with details provided in Table 1

Thin sections of cells cultured under low-oxygen conditions, which are permissive for growth of FnrL null mutant bacteria but also support some FnrL regulatory activity (Roh and Kaplan 2002), were examined using TEM (Fig. 4A). In contrast to the typical high density of ICM observed in the thin sections of wild type cells, approximately 5–10 ICM-like structures per cell were seen in the sections of the *fnrL* null mutant JZ1678. While the number of these structures is approximately the same as that seen in sections of the PrrA^−^ mutant bacteria cultured under low-oxygen conditions (Fig. 1A), spectral complexes are detectable in cells lacking FnrL (Zeilstra-Ryalls et al. 1997), which correlates with regulation of different sets of genes by these two transcription factors (Gomelsky and Zeilstra-Ryalls 2013), even though both are indispensable for phototrophic growth.

Although both *R. sphaeroides* and *R. capsulatus* require FnrL for anaerobic–dark growth with DMSO, *R. capsulatus* FnrL^−^ mutant bacteria are capable of phototrophic growth, and spectral complex levels are unaffected by the absence of FnrL (Zeilstra-Ryalls et al. 1997). The ultrastructure of *R. capsulatus fnrL* null mutant bacteria, strains RGK295 and 296 (Table 1), was evaluated by preparing thin sections of cells cultured under low-oxygen conditions and examining them using TEM (Fig. 4B). In contrast to the abnormal appearance of *R. sphaeroides* FnrL^−^ mutant bacterial cell membranes (Fig. 4A), the membrane morphology of *R. capsulatus* FnrL^−^ bacteria appeared similar to the FnrL^+^ parent strain SB1003 (Table 1). Therefore, for *R. capsulatus*, the absence of FnrL apparently did not affect ICM formation. This predicts that there are genes necessary for ICM development in *R. sphaeroides* whose transcription is regulated by FnrL, but that in *R. capsulatus* are not FnrL-dependent (or absent).

## Discussion

Transcriptomic and proteomic investigations have provided insights into regulatory events that are mediated by PrrA, PpsR, and FnrL as *R. sphaeroides* responds to changes in oxygen availability (reviewed in Gomelsky and Zeilstra-Ryalls 2013). Spectral analysis has also been a useful tool in studying the roles of these DNA binding proteins in the formation of pigment–protein complexes. This study of membrane structure in mutants missing one or more of these global regulators has provided a different perspective and has generated new findings.

Based on the TEM results, the *prr* genes are required for normal ICM formation. An unanticipated and novel discovery made during these studies was the ultrastructural differences of low-oxygen cells with defective *prrA* genes versus those in which the entire *prr* gene cluster is absent. The presence of ICM-like structures in *prrA* null mutant bacteria and their absence in *prrBCA* ^−^ bacteria suggests that PrrB and/or PrrC may participate in regulation of genes associated with ICM formation that does not involve PrrA activity. To what degree these ICM-like structures resemble true ICM will require an in-depth analysis of their molecular composition. While for cells cultured anaerobically in the dark transcriptomic and proteomic data are available, which could be used as a guide to direct us to potentially important genes regulated by PrrA involved in ICM formation, there is currently no similar data available at the genome wide level for PrrB or C, nor for cells grown under low-oxygen conditions. Before this investigation, the presence of such structures, and so the need for such information was not evident, since other methods used to evaluate the physiological status of *R. sphaeroides*, such as comparisons of growth rates or even spectral analyses, gave no indication that there were any differences between cells lacking *prrA* alone versus those lacking all three *prr* genes under any condition.

It is possible that the ultrastructure differences might be explained by cross-talk or branched regulation between PrrB and a non-cognate response regulatory protein. Such a regulator must be able to recognize at least a subset of PrrA targets and, to be consistent with the results presented here, it must be present in cells grown under low-oxygen conditions but absent in cells grown anaerobically in the dark with DMSO. Interestingly, there is evidence suggesting that PrrA regulation may be affected by kinase activity of the non-cognate sensor protein HupT (Gomelsky and Kaplan 1995), which is a histidine kinase for hydrogen uptake. However, to our knowledge, there are no prior reports of PrrB promiscuity with respect to other response regulators.

The model of the hierarchical regulation of genes involving PpsR and PrrA proposes that the inability of PrrA mutant bacteria to grow phototrophically is not due to the lack of PrrA-mediated activation of PS genes; rather, it is the inability to anti-repress PpsR-regulated genes (Gomelsky et al. 2008). The presence of aberrant structures in bacteria lacking both PrrA and PpsR suggests this model is incomplete, and that there may be genes regulated by PrrA, but not by PpsR, that are required for normal ICM development.

While the essential PS genes of *R. sphaeroides* 2.4.1 are little changed in their transcription levels by the presence versus the absence of FnrL (reviewed in Gomelsky and Zeilstra-Ryalls 2013), *fnrL* null mutant bacteria are nevertheless unable to form normal ICM. This study has identified a potential route to the identification of FnrL-dependent genes other than PS genes that are required for ICM formation, since unlike *R. sphaeroides* FnrL mutants, *R. capsulatus* FnrL mutants are unaltered in their ability to grow phototrophically (Zeilstra-Ryalls et al. 1997), and the ultrastructure of the *R. capsulatus* ICM appeared normal. The prediction is that there are genes necessary for the differentiation process to take place that are regulated by FnrL in *R. sphaeroides* but not in *R. capsulatus*.
